# Incidence of Mortality and Complications in High-Risk Pulmonary Embolism: A Systematic Review and Meta-Analysis

**DOI:** 10.1016/j.jscai.2022.100548

**Published:** 2023-01-27

**Authors:** Mitchell J. Silver, Jay Giri, Áine Duffy, Wissam A. Jaber, Sameer Khandhar, Kenneth Ouriel, Catalin Toma, Thomas Tu, James M. Horowitz

**Affiliations:** a Department of Cardiovascular Medicine, Ohio Health Heart and Vascular, Columbus, Ohio; b Penn Cardiovascular Outcomes, Quality and Evaluative Research Center, Cardiovascular Medicine Division, University of Pennsylvania, Philadelphia, Pennsylvania; c North American Science Associates, LLC, New York, New York; d Division of Cardiology, Emory University School of Medicine, Atlanta, Georgia; e Division of Cardiovascular Medicine, Penn Presbyterian Medical Center, Philadelphia, Pennsylvania; f Heart and Vascular Institute, University of Pittsburgh Medical Center, Pittsburgh, Pennsylvania; g Inari Medical, Irvine, California; h Division of Cardiology, NYU Langone Health, New York, New York

**Keywords:** high-risk, massive, mechanical thrombectomy, pulmonary embolism, thromboembolectomy, thrombolysis

## Abstract

**Background:**

The relationship between the early hemodynamic consequences of acute pulmonary embolism (PE) and short-term morbidity and mortality has long been recognized. The mortality incidence and other complications after high-risk (massive) PE, the most severe category of the disease, are summarized in this meta-analysis.

**Methods:**

A systematic review and meta-analysis of studies reporting on patients with massive PE indexed by PubMed and the Cochrane Library over a 10-year period (2010-2020) was conducted. Studies with adequate information to specify a cohort of patients with high-risk PE defined by the American Heart Association and European Society of Cardiology criteria and their clinical outcomes were included. Incidences were calculated as weighted averages with 95% CIs.

**Results:**

A total of 27 publications spanning 1517 patients were identified that met the search criteria for high-risk PE. In-hospital all-cause mortality averaged 28.3% (95% CI, 20.9%-37.0%) in patients at high risk, comparable to the 30-day all-cause mortality of 30.2% (95% CI, 22.3%-39.6%). In-hospital major bleeding was 13.8% (95% CI, 9.3%-20.0%), and intracranial hemorrhage was reported in 3.6% (95% CI, 2.2%-5.9%). The risk of bias in publications was graded as low-to-moderate, with substantial heterogeneity among the studies.

**Conclusions:**

This systematic review and meta-analysis provided low-quality to moderate-quality evidence documenting mortality, major bleeding, and other complications in patients meeting the American Heart Association and European Society of Cardiology criteria for high-risk PE. This information was used to inform the design of the FLowTriever for Acute Massive Pulmonary Embolism (FLAME) study (NCT04795167), a study evaluating an advanced therapy for patients with high-risk PE.

## Highlights

• Systematic review, meta-analysis of high-risk (massive) pulmonary embolism outcomes
• In-hospital and 30-day mortality averaged 28.3% and 30.2% in high-risk patients
• In-hospital major bleeding and intracranial hemorrhage rates were 13.8% and 3.6%
• Insufficient data for breakdown by treatment type (thrombectomy, thrombolysis)

## Central Illustration

**Figure:**
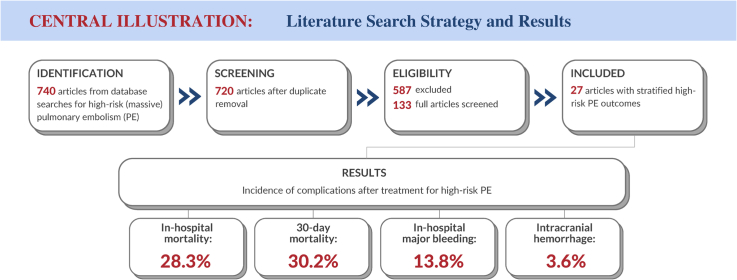

## Introduction

Pulmonary embolism (PE) is associated with up to 300,000 deaths annually in the United States and represents the third leading cause of cardiovascular death, after myocardial infarction and stroke.^1^ The mortality of PE varies along the spectrum of patients with minor PE through those who present with hemodynamic compromise.^2^, ^3^, ^4^ Experts recommend tailoring treatment with anticoagulation, thrombolytic therapy, endovascular interventions, or surgical thrombectomy based on risk stratification.^4^, ^5^, ^6^, ^7^ Anticoagulation without advanced treatment can be considered for certain hemodynamically stable patients with PE without evidence of right ventricular dysfunction or myocardial tissue injury, whereas the rapid institution of reperfusion therapies such as systemic or catheter-directed thrombolysis, mechanical thrombectomy, or surgical thrombectomy may be more appropriate for those with hypotension, right ventricular dysfunction, or end-organ hypoperfusion.^8^^,^^9^

There is general agreement that therapy of PE should be severity specific.^4^^,^^10^ Improved outcome is contingent upon early recognition, risk stratification, and rapid treatment tailored to severity. Mortality for high-risk PE is a particularly pressing public health concern. Past analyses used various criteria to designate high-risk status, including radiographic thrombus burden and various clinical presentation characteristics. It is unclear what the clinical outcomes and complication incidences are among uniformly defined patients with high-risk PE across a range of modern cohorts. The 2019 publications from the American Heart Association (AHA) and European Society of Cardiology (ESC) provided standardized definitions for the 3 categories of severity: low-risk, intermediate-risk, and high-risk.^11^^,^^12^ High-risk PE, also known as massive PE (AHA),^13^ is defined by systolic blood pressure of <90 mm Hg or a drop of >40 mm Hg lasting at least 15 minutes or when vasopressor support is needed (AHA and ESC) or when a cardiac arrest has occurred (ESC).^11^^,^^12^

Using these modern consensus definitions for high-risk PE, we performed a systematic literature review and meta-analysis to ascertain the outcome of contemporary treatment strategies, tabulating mortality, bleeding, and clinical deterioration. This information provided benchmarks for the design of the FLowTriever for Acute Massive Pulmonary Embolism (FLAME) study (NCT04795167), which began enrollment in 2021 to evaluate an advanced therapy for high-risk PE. It may also be used in the analysis of future clinical trials and the assessment of real-world outcomes at the local, regional, and global levels among this challenging patient population.

## Methods

### Objectives

The objective was to collect and analyze data on outcomes in patients presenting with high-risk PE, using the 2019 AHA and ESC criteria to (1) define the high-risk cohort of patients in prior studies and (2) employ uniform definitions for the assessment of important safety and effectiveness end points.

### Preparation of the systematic review and meta-analysis

A protocol-driven literature review was conducted according to the systematic methodology outlined in the Preferred Reporting Items for Systematic Reviews and Meta-Analyses statement.^14^ The protocol was prospectively developed prior to initiating the search. The review and its strategy were registered with PROSPERO (CRD42020219695).

### Search strategy

Two databases were searched for articles published over the 10-year period between April 2010 and March 2020. The end date of March 2020 was selected because this literature review was performed in part to support the aforementioned FLAME study, the design for which was finalized in 2020 and enrollment for which began in 2021. PubMed (US National Library of Medicine) was the primary database, and the Cochrane Library was the secondary database to provide comprehensive coverage of the literature. Publications appearing in the bibliographies of review articles were retrieved by the search. Reviewers independently screened the titles and abstracts of each retrieved article using the DistillerSR application (Evidence Partners).^15^^,^^16^ Following the identification of candidate publications, full-text articles were assessed for eligibility by 2 reviewers (A.D. and K.O.). Disagreements on inclusion were resolved by consensus or, when consensus could not be reached, through a teleconference with all authors.

### Eligibility criteria for publications and inclusion of end points

The primary reviewer (A.D.) performed the initial search. Inclusion criteria were predetermined by all authors. Publications were eligible for inclusion if they were published over the prespecified 10-year timeframe, were not review articles, identified the inclusion criteria with specificity to permit determination of the AHA/ESC criteria for high-risk PE, enabled subcategorization of relevant outcomes to patients at high risk when more than 1 PE severity category was included, and in which end points were defined with adequate precision to meet the prespecified criteria for uniformity. Studies with <10 patients were excluded, as were publications that were not published in English. Publications that adequately met these criteria for some but not all end points were included in the overall review, but those end points that did not meet prespecified criteria were excluded from the analysis. Exclusion of 1 or more end points from some publications due to unreported data resulted in varying denominators for each end point. In addition, end points where the timeframe over which the end point event occurred were not specified, uncertain, or outside of the intervals specified for this review were excluded. For cases in which one publication reported separate cohorts treated with different modalities, the cohorts were tabulated separately. The secondary reviewer (K.O.) reviewed the list of publications for inclusion, with additions or exclusions from articles identified from bibliographies of review articles or otherwise identified by the authors.

### Outcome measures

Mortality outcomes and secondary end points in the publications that met the inclusion criteria were assessed. Mortality outcome measures included in-hospital all-cause mortality (ACM) and ACM through 30 days as reported in the study. Mortality from PE was also assessed in those publications in which it was specified through 30 days.

Secondary outcome measures included the incidence of bailout therapy for initial treatment failure (eg, from thrombolysis to surgical thrombectomy), clinical deterioration, major bleeding defined as Bleeding Academic Research Consortium (BARC) 3b or greater, study-defined major bleeding (irrespective of BARC grade), intracranial hemorrhage (ICH), stroke, renal dysfunction, and sepsis. BARC 3b was defined as overt bleeding with a hemoglobin drop of at least 5 g/dL, cardiac tamponade, bleeding requiring surgical intervention, or bleeding requiring intravenous vasoactive agents.^17^ Secondary end point incidences were, by and large, reported through discharge from the index PE hospitalization.

### Data extraction methodology

Data were extracted by the primary reviewer (A.D.) in an independent and unblinded fashion using a prespecified search protocol (Table 1). Abstracted data were collected at the publication level and the end point level. At the publication level, the characteristics of each study were tabulated, including study size, study design (arms, randomization), geography, years of treatment, duration of follow-up, and age and sex of the patients. The index hospitalization was defined as the initial hospitalization for PE, as specified in the publications. The primary review abstracted available end points from each publication. All datapoints were verified by the secondary reviewer (K.O.) after a subsequent review of each publication. Any discrepancies between the reviewers were resolved with discussion or, when necessary, by consensus with a teleconference with the coauthors. Further, the coauthors met as a group on several occasions where the end points were reviewed for the opportunity to double-check apparent outliers and to determine whether there was any overlap among studies.

**Table 1:** Boolean search strategy.

Step	Search string	Results yielded	
		PubMed	Cochrane
1	Pulmonary embolism	51,539	3203
2	#1 AND (massive or “hemodynamically unstable” or “high risk”)	6116	510
3	#2 AND (thrombolysis OR embolectomy OR thrombectomy)	1509	68
4	#3 AND Filters: published in the last 10 years; humans^a^	675	65

		Overall results
Total		740
Duplicates		20
Unique publications		720

a “Humans” filter applies only to the MEDLINE (PubMed) database.

### Quality assessment, potential publication bias, and heterogeneity

Retrieved publications were assessed for the quality of evidence and the risk of bias, and the level of certainty was rated using the Grading of Recommendations, Assessment, Development and Evaluations (GRADE) guidelines.^18^ Each study was subjectively ranked as very low, low, moderate, or high quality by the primary and secondary reviewers, and discordance was resolved with discussion. The aggregate quality of evidence for the review was assessed by the authors after reviewing the quality, risk of bias, and certainty for each of the individual included publications.

Heterogeneity was assessed using the I^2^ statistic for heterogeneity.^19^^,^^20^ The level of heterogeneity was based on the I^2^ statistic and was graded as either low (0%-25%), moderate (26%-50%), or high (>50%).^21^

### Statistical analysis

For each study included in the final analysis, descriptive data were collected on study characteristics. Individual end point data were assessed as crude proportions expressed as the ratio of the reported frequency of an event to the number of observations excluding missing values. The logit transformation for proportions was used, and the DerSimonian and Laird method for random effects (a variation on the inverse-variance method) was used to produce weighted average event incidences and associated 95% CIs.^22^ Statistical analyses were performed with the Comprehensive Meta-Analysis (CMA) software package (version 3.3.070, Biostat).

## Results

### Selection of publications for analysis

**Figure 1:**
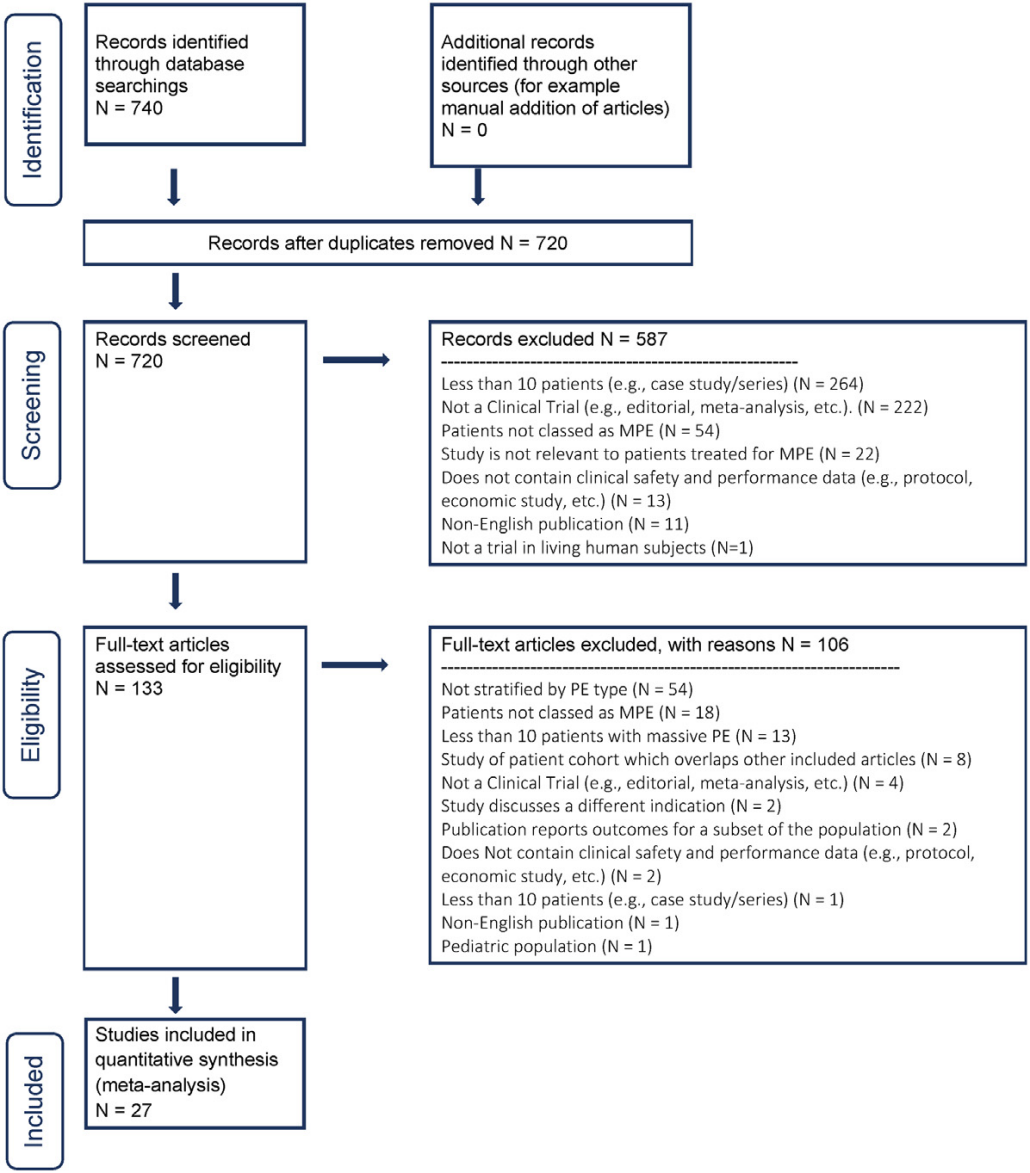
**Preferred Reporting Items for Systematic Reviews and Meta-Analyses flow diagram demonstrating the selection process for included publications.** The arrows indicate the direction of workflow. The numbers indicate the number of publications remaining at each step in the process. MPE, massive pulmonary embolism; PE, pulmonary embolism.

The selection results are summarized in Figure 1. There were 740 abstracts identified by the initial search and screened for inclusion, and 20 of these abstracts were duplicates, leaving 720 unique abstracts identified for review. Among these, 587 studies were excluded for various reasons, the most common being a small sample size (<10 patients), leaving 133 publications identified for full-text review. After a review of the full-text publications by the reviewers, 106 studies were excluded. The most common reason for exclusion was the inability to assess a cohort of patients meeting the definition of high-risk PE, a limitation in 54 publications, leaving 27 articles in the final set of publications that formed the basis of the current systematic review and meta-analysis.^23^, ^24^, ^25^, ^26^, ^27^, ^28^, ^29^, ^30^, ^31^, ^32^, ^33^, ^34^, ^35^, ^36^, ^37^, ^38^, ^39^, ^40^, ^41^, ^42^, ^43^, ^44^, ^45^, ^46^, ^47^, ^48^, ^49^

### Characteristics of the publications

Among the 27 included publications, 22 (81.5%) were retrospective and 5 (18.5%) were prospective; 22 studies were performed at a single investigational site, whereas 5 were multicenter studies. All but 1 study was nonrandomized. There were 21 single-arm studies (77.7%) and 6 (22.2%) studies that had >1 treatment cohort evaluated. Studies were performed in the United States in 8 (29.6%) publications, Asia-Pacific in 8 (29.6%), Europe in 9 (33.3%), South America in 1 (3.7%), and both Europe and the United States in 1 (3.7%).

Although the publications were all after 2010, treatment was performed between 1992 and 2017; only 3 studies (11.1%) included patients treated before the year 2000. Follow-up after the index treatment was a median of 14.5 months (range, 1-48 months) in the studies.

### Characteristics of the study participants

A total of 1517 patients were included in the quantitative analysis of the 27 studies that met the criteria for this review. The age of patients in the series ranged between 45 and 80 years, and on average, 41% (range, 17%-56%) of the patients were male. The therapies used in the studies of high-risk PE included anticoagulation alone, systemic thrombolysis, catheter-directed thrombolysis, mechanical thrombectomy, and open surgical thrombectomy. Extracorporeal membrane oxygenation was a primary treatment strategy in 1 cohort and was used selectively as an adjunct to other interventions for patients with hemodynamic compromise. More than 1 therapy was used in most publications. Outcomes were analyzed as independent study cohorts for publications that reported therapeutic subgroups separately. However, a comparative analysis of different therapeutic modalities was not an objective of the current review and was not performed.

### Mortality outcomes

**Figure 2:**
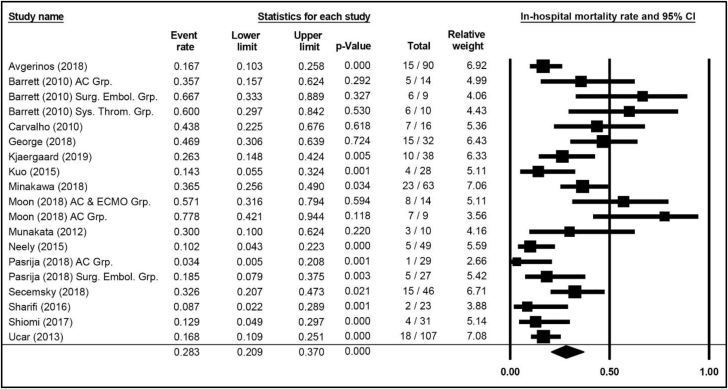
**In-hospital mortality weighted average, 95% CIs, and forest plot for patients with high-risk pulmonary embolism.** Additional information is presented in Supplemental Table S3. AC Grp., anticoagulation group; AC & ECMO, anticoagulation and extracorporeal membrane oxygenation group; Surg. Embol. Grp., surgical embolization group; Sys. Throm. Grp., systemic thrombolysis group.

**Figure 3:**
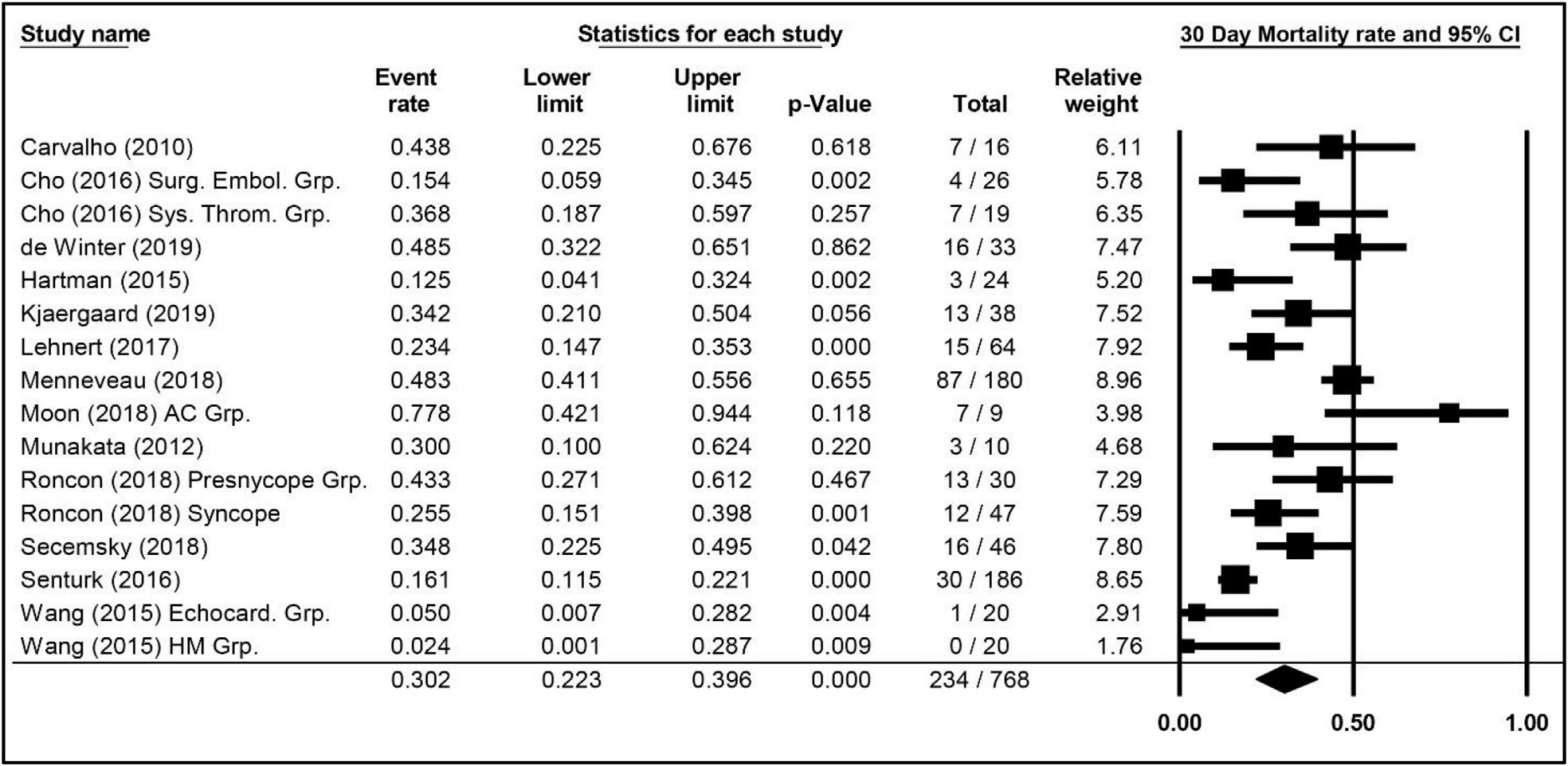
**Mortality weighted average through 30 days, 95% CIs, and forest plot for patients with high-risk pulmonary embolism.** Additional information is presented in Supplemental Table S3. AC Grp., anticoagulation group; Echocard. Grp., echocardiology group; HM Grp., hemodynamic monitoring group; Surg. Embol. Grp., Surgical Embolization Group; Sys. Throm. Grp., systemic thrombolysis group.

Mortality outcomes for in-hospital, 30-day, and PE-related outcomes (through follow-up) are summarized in Supplemental Table S1. In-hospital ACM was reported in 15 publications comprising 19 therapeutic cohorts and 631 patients, with a weighted average of 28.3% (95% CI, 20.9%-37.0%; Figure 2). ACM through 30 days was reported in 13 publications comprising 16 cohorts with 782 patients, occurring in 30.2% (95% CI, 22.3%-39.6%; Figure 3). Mortality attributed to PE was specified in 3 publications and 6 cohorts with 118 patients, with an incidence of 38.6% (95% CI, 24.4%-55.0%) of the observed mortality (Central Illustration).

### Secondary outcomes

Outcomes for secondary, nonmortality outcomes are summarized in Supplemental Table S2. Crossover to an alternative thrombus removal therapy beyond the primary therapeutic modality was specified in 3 publications with 4 cohorts, reporting crossover in 28 of 92 patients, accounting for a weighted average of 30.3% (95% CI, 15.5%-50.7%). Clinical deterioration within the first 24 hours after the index PE was reported in only 1 publication, in which the observed frequency was 5 of 32 patients (15.6%; 95% CI, 6.7%-32.5%).

**Figure 4:**
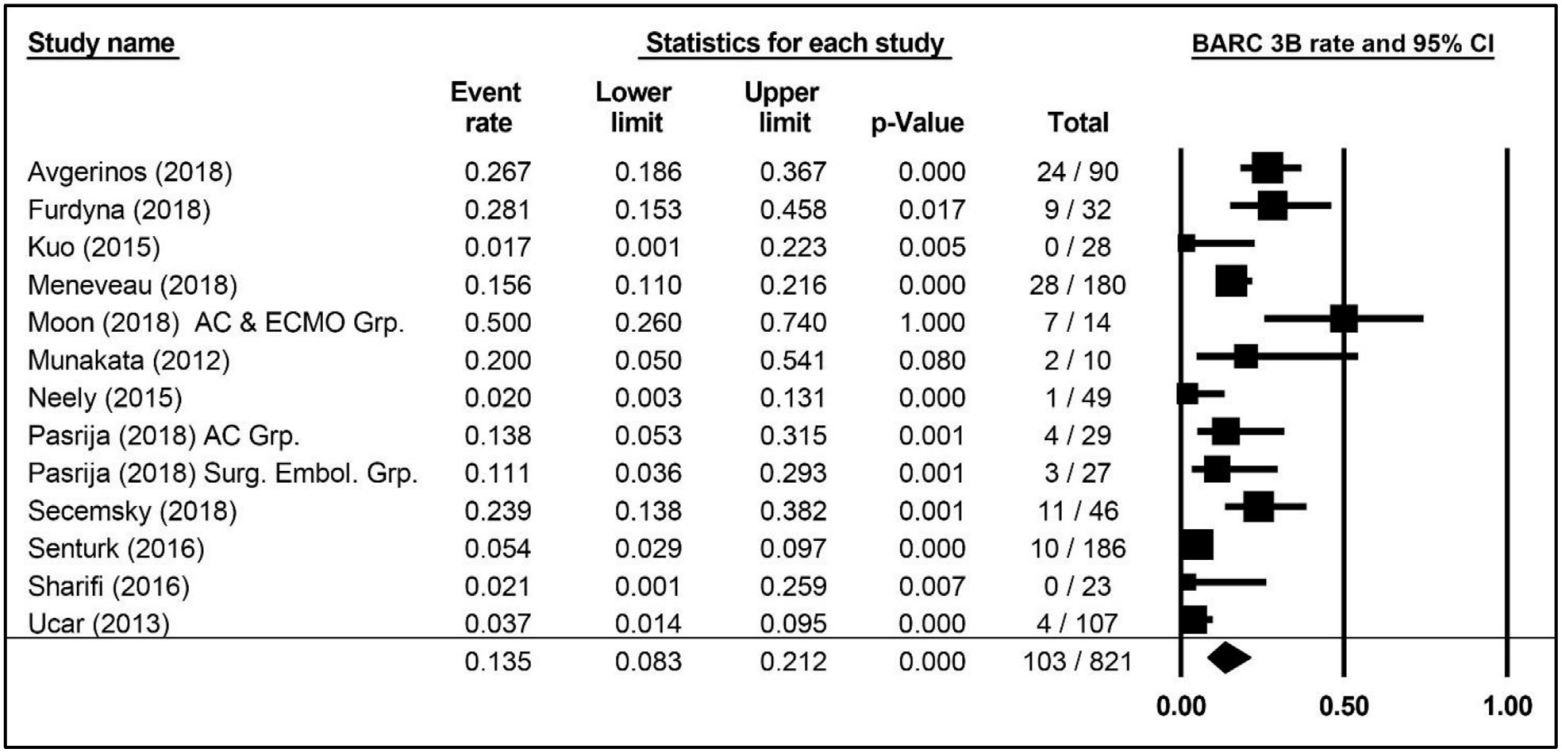
**Weighted averages, 95% CIs, and forest plot of publications specifying major (BARC 3b) bleeding incidence in patients with high-risk pulmonary embolism.** Additional information is presented in Supplemental Table S1. AC Grp., anticoagulation group; AC & ECMO, anticoagulation and extracorporeal membrane oxygenation group; BARC, Bleeding Academic Research Consortium; Surg. Embol. Grp., surgical embolization group.

**Figure 5:**
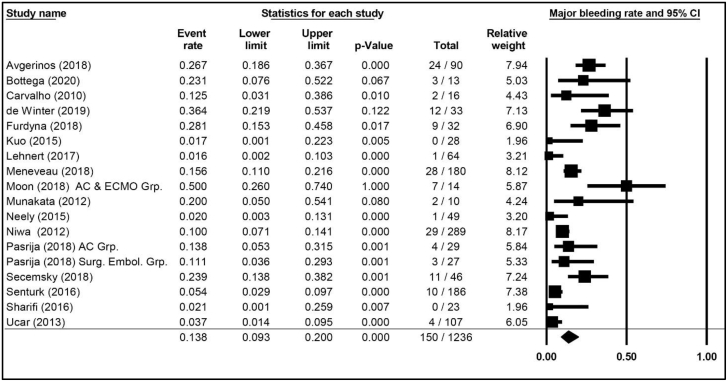
**Weighted averages, 95% CIs, and forest plot for major bleeding (irrespective of BARC) in patients with high-risk pulmonary embolism.** Additional information is presented in Supplemental Table S1. AC Grp., anticoagulation group; AC & ECMO, anticoagulation and extracorporeal membrane oxygenation group; BARC, Bleeding Academic Research Consortium; Surg. Embol. Grp., surgical embolization group.

Major (BARC 3b or greater) bleeding was reported in 12 publications comprising 13 cohorts and 821 patients, with a weighted average risk of 13.5% (95% CI, 8.3%-21.2%; Figure 4). The weighted average for study-defined major bleeding was 13.8% (95% CI, 9.3%-20.0%; Figure 5), reported in 17 publications with 18 cohorts and 1236 patients. ICH was reported in 4 publications comprising 4 cohorts and 453 patients and accounting for an ICH weighted average of 3.6% (95% CI, 2.2%-5.9%).

**Central Illustration:**
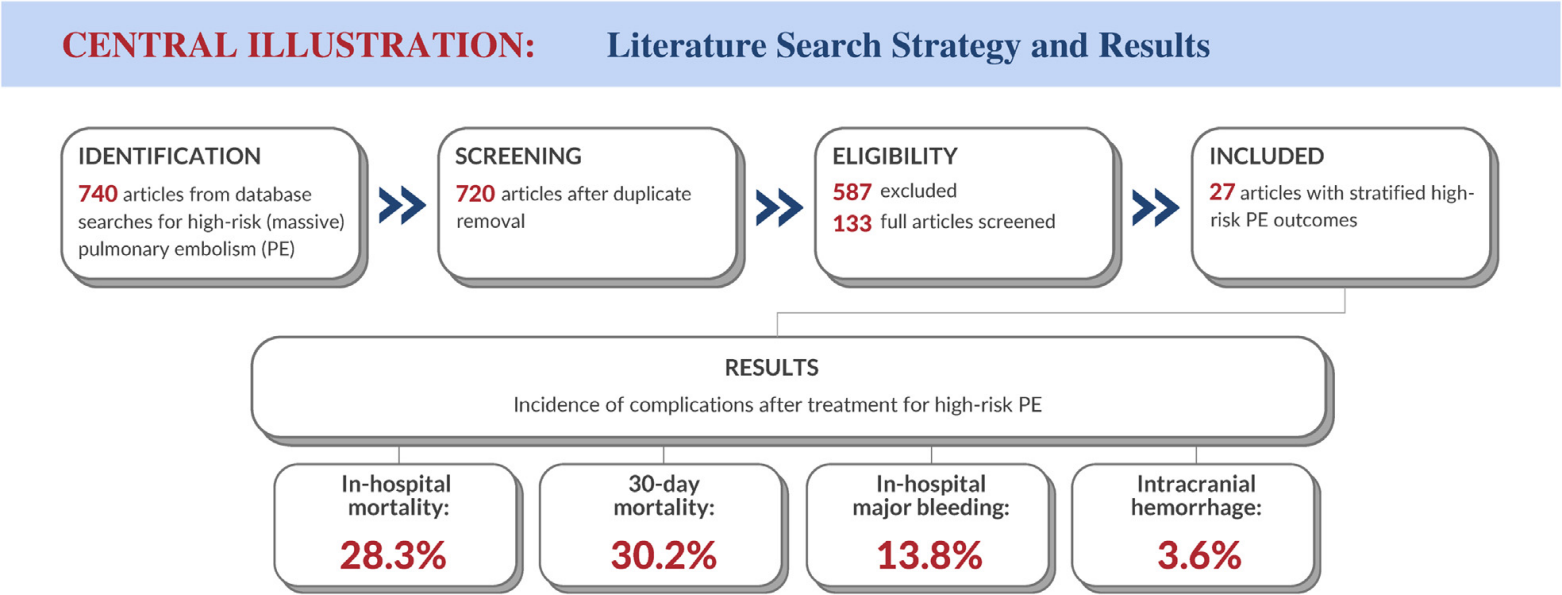
**Literaturesearch strategy and results.**

Sepsis after the index PE was reported in 2 publications comprising 3 cohorts and 105 patients, with an incidence of 4.9% (95% CI, 2.1%-11.3%). Renal dysfunction was reported in 3 publications, 4 cohorts, and 121 patients, with an incidence of 11.4% (95% CI, 6.7%-18.7%). Stroke was reported in 5 publications, 6 cohorts, and 240 patients, with an incidence of 5.6% (95% CI, 2.2%-13.5%). The publications did not uniformly subcategorize stroke into hemorrhagic and nonhemorrhagic events, accounting for possible overlap between ICH and stroke end points.

### Quality of evidence and publication bias

The overall quality of evidence in this review was graded as “low” to “moderate” per the GRADE classification. The relatively small study size of the reviewed publications contributed to this rating as did the observational nature of most of the studies. Only 1 study was randomized, and none were blinded. There appeared to be moderate-to-high heterogeneity among the studies. I^2^ values for mortality outcomes ranged from 54.5% for PE-related mortality to 79.5% for 30-day ACM. There were low levels of heterogeneity for the secondary outcome measures of clinical deterioration, ICH, sepsis, and renal dysfunction, with I^2^ values of 0.0% for each end point. By contrast, there were high levels of heterogeneity for bleeding end points, with I^2^ values of 79.5% for major bleeding (BARC 3b or greater) and 78.7% for major bleeding (irrespective of BARC grade).

## Discussion

Pulmonary embolism is a deadly disease, with a 30-day mortality incidence ranging between 9% and 44% for centers participating in the US multicenter Pulmonary Embolism Response Team (PERT) Consortium.^50^ Such variability has been common in the literature and can be explained by differences in the populations studied with vastly different mortality incidence that, in part, depends on the thrombus burden and the frailty of the patient.^4^^,^^5^^,^^51^ High-risk PE, also known as massive PE, carries a mortality incidence reported to average approximately 30% or more.^11^^,^^13^^,^^52^ These estimates also suffer from a lack of uniformity in risk category definition and are limited by heterogeneity and a lack of randomized data. Nonetheless, current literature documents high mortality that is only modestly improved with advanced therapies such as catheter-directed thrombolysis, ultrasound-accelerated thrombolysis, and open surgical thrombectomy.^24^^,^^33^^,^^37^

The lack of uniformity in reporting was addressed in the AHA and ESC 2019 scientific statements.^11^^,^^12^ These 2 documents set a standard for definitions to stratify patients into the 3 PE risk categories: low-risk, intermediate-risk, and high-risk. The ESC guidelines further stratify the intermediate-risk group into intermediate-high and intermediate-low. Using the AHA and ESC definitions, the current review estimated the incidence of mortality and other important end points in the subset of patients with well-defined high-risk PE. To the extent possible, mortality and other important outcomes were aligned between publications. A prespecified search strategy was developed and executed, identifying 27 relevant high-risk PE articles published over the last decade.

Bleeding complications of PE were reasonably well documented in the publications. Classification of major bleeding defined by BARC 3b or greater or equivalent was documented in 12 publications with an incidence of approximately 14%. Major bleeding not specified by BARC was similar, reported in 14% of patients. ICH was reported in only 4 publications but with approximately 450 patients, at an incidence of 3.6%. The robustness of the ICH estimate was limited by studies that might not have specifically reported ICH when none occurred, and the calculated estimate may be an overestimate. The incidence of sepsis, renal insufficiency, and stroke were also evaluated in the review, with weighted averages of 4.9%, 11.4%, and 5.6%. These complications, however, were reported in a small number of publications and patients.

When measured in well-defined populations of patients with high-risk PE, the mortality incidence averaged approximately 30%, both in-hospital and through 30 days. Where individual treatment modalities were reported, the incidence of in-hospital mortality was similar among groups (25% for anticoagulation,^23^^,^^32^^,^^36^ 22% for systematic thrombolysis,^23^^,^^38^^,^^40^^,^^42^ 23% for catheter-directed thrombolysis,^22^^,^^28^^,^^30^ and 26% for surgical embolectomy^23^^,^^32^^,^^36^). The 30-day incidence of ACM was 78% in 1 study that reported on anticoagulation in this analysis.^32^ The other treatment modalities showed a similar lower incidence of 30-day ACM when compared with the anticoagulation group (35% for systemic thrombolysis,^25^^,^^38^ 30% for catheter-directed thrombolysis,^33^ and 26% for surgical embolectomy^24^). Clinical deterioration within the first 24 hours after initiation of therapy was reported in only 1 publication, rendering the 16% estimation imprecise. Crossover to alternate therapy occurred in approximately 30% of patients, likely closely related to clinical deterioration because additional therapy is generally indicated for initial treatment failure. Still, this estimate was based on only 3 publications.

This systemic review and meta-analysis had several limitations. There were few prospectively designed studies that met the search criteria, only 5 of the 27 publications. As a consequence of the low number of prospective studies included, the GRADE classifications were in the low-to-moderate range, an effect of the preponderance of single-arm, often retrospective studies. There were missing values in each of the publications that were included in the review, which can create bias to the extent that missingness was not at random. Heterogeneity of the studies was another limitation of this analysis. Heterogeneity of many of the end points was high, particularly for the mortality and bleeding end points. Other limitations were a lack of patient-level data and a lack of per-treatment analysis. Lastly, there was only 1 randomized study included in the analysis. However, this is not a limitation per se, as the dearth of randomized trials is explained by the study populations of hemodynamically compromised patients presenting for urgent treatment. As noted in the AHA scientific statement on PE, flexibility is essential in managing this critically ill population, and a randomized design would likely lead to many crossovers, thereby biasing results.^11^ The rarity of randomized trials is understandable in the emergency setting of high-risk PE, in which patient consent and almost all other operational aspects of such trials are difficult.

In conclusion, this systematic review and meta-analysis provides low-to-moderate quality evidence per GRADE criteria documenting high event incidences (mortality, bleeding, and other complications) in patients meeting the AHA and ESC criteria for high-risk PE. It is understandable that high-quality evidence from randomized studies is not available owing to the difficulty in conducting randomized analyses in patients presenting urgently with hemodynamic compromise. For this reason, future multicenter registries that are able to capture all sequential high-risk PEs will be very important. Morbidity and mortality remain high in this challenging group of patients. The information from this review was used in the planning of the FLAME study to evaluate an advanced therapy for high-risk PE and will be of use in the planning of future clinical trials of new therapies for patients with high-risk PE, both for the choice of appropriate end points and in the estimation of historical incidences for performance goals and sample size calculations.
