# Supplementary material for: Incidence of Mortality and Complications in High-Risk Pulmonary Embolism: A Systematic Review and Meta-Analysis
Source: J Soc Cardiovasc Angiogr Interv. 2023 Jan 27;2(1):100548. doi: 10.1016/j.jscai.2022.100548 (PMC11308857; doi:10.1016/j.jscai.2022.100548)
Supplement: Supplemental Tables S1 to S3 [file mmc1.docx]

| Supplemental Table 1. Publications Included in the Review- ON-LINE ONLY | | | | | | | | | |
| --- | --- | --- | --- | --- | --- | --- | --- | --- | --- |
| **Publication** | **Patients** | **High-Risk Definition** | **Treatment** | **Study Design** | **Country** | **Follow-Up *** | **Treatment Period** | **Age†** | **Male (%)** |
| Avgerinos ED, Abou Ali AN, Liang NL, Rivera-Lebron B, Toma C, Maholic R, et al. Catheter-directed interventions compared with systemic thrombolysis achieve improved ventricular function recovery at a potentially lower complication rate for acute pulmonary embolism. Journal of vascular surgery Venous and lymphatic disorders. 2018;6(4):425-32. 10.1016/j.jvsv.2017.12.058 | 90 | Sustained hypotension for at least 15 minutes or requiring vasopressors. | Catheter directed treatment including thrombolysis and mechanical thrombectomy (high-risk group). | Retrospective, single-arm, single-center, non-randomized study | US | 1 | 2006-2016 | NS | NS |
| Barrett NA, Byrne A, Delaney A, Hibbert M, Ramakrishnan N. Management of massive pulmonary embolism: a retrospective single-centre cohort study. Critical care and resuscitation : journal of the Australasian Academy of Critical Care Medicine. 2010;12(4):242-7. | 33 | Hypotension (systolic blood pressure <90mmHg) and shock. | Thrombolysis (N=10), surgical embolectomy (N=9), or heparin (N=14). | Retrospective, three-arm, single-center, non-randomized study | Australia | NS | 1996-2006 | NS | NS |
| Bottega TS, Vier MG, Baldiaserotto H, Oliveira EP, Diaz CLM, Fernandes CJ. Thrombolysis in acute pulmonary embolism. Revista da Associacao Medica Brasileira (1992). 2020;66(3):263-7. 10.1590/1806-9282.66.3.263 | 13 | Diagnosis of high-risk PTE according to the classification suggested by the consensus of the European Society of Cardiology /European Respiratory Society. | Thrombolysis | Retrospective, single-arm, single-center, non-randomized study | Brazil | NS | 2014-2016 | NS | NS |
| Carvalho EM, Macedo FI, Panos AL, Ricci M, Salerno TA. Pulmonary embolectomy: recommendation for early surgical intervention. Journal of cardiac surgery. 2010;25(3):261-6. 10.1111/j.1540-8191.2009.00986.x | 16 | Occlusion of the pulmonary artery that exceeds 50% of its cross-sectional area, resulting in progressive hemodynamic compromise. | Surgical pulmonary embolectomy, systemic heparin anticoagulation. | Retrospective, single-arm, single-center, non-randomized study | US | 48±38 | 2000-2009 | 45±17 | 9 |
| Cho YH, Sung K, Kim WS, Jeong DS, Lee YT, Park PW, et al. Management of acute massive pulmonary embolism: Is surgical embolectomy inferior to thrombolysis? International journal of cardiology. 2016;203:579-83. 10.1016/j.ijcard.2015.10.223 | 45 | Sustained hypotension, pulselessness, or persistent profound bradycardia with signs or symptoms of shock in the presence of a newly developed thrombus in the common trunk or right or left main pulmonary artery. | Thrombolysis or surgical embolectomy. | Retrospective, single-arm, single-center, non-randomized study | Korea | 17 (IQR: 53–74) | 2000-2013 | 68 (IQR: 53–74) | 17 |
| Corsi F, Lebreton G, Brechot N, Hekimian G, Nieszkowska A, Trouillet JL, et al. Life-threatening massive pulmonary embolism rescued by venoarterial-extracorporeal membrane oxygenation. Critical care (London, England). 2017;21(1):76. 10.1186/s13054-017-1655-8 | 17 | Acute refractory cardiovascular failure, defined as evidence of tissue hypoxia concomitant with adequate intravascular volume status; severely diminished RV or left ventricular ejection fraction (RV/LVEF); low cardiac index (≤2.1 /min/m^2^); sustained hypotension despite high-dose catecholamine infusion. | Thrombectomy, systemic fibrinolytic therapy, embolectomy or thromboaspiration, followed by ECMO. | Retrospective, single-arm, single-center, non-randomized study | France | 19 (4-74) | 2006-2015 | 51 (18-70) | 6 |
| de Winter MA, Hart EA, van den Heuvel DAF, Moelker A, Lely RJ, Kaasjager KAH, et al. Local Ultrasound-Facilitated Thrombolysis in High-Risk Pulmonary Embolism: First Dutch Experience. Cardiovascular and interventional radiology. 2019;42(7):962-9. 10.1007/s00270-019-02200-1 | 33 | Shock or hypotension (systolic blood pressure <100 mmHg or a decline of >40 mmHg. | Ultrasound-facilitated catheter-directed thrombolysis, systemic thrombolysis | Retrospective, single-arm, multi-center, non-randomized study | Netherlands | 3 | 2010-2017 | 63 (IQR: 51-71) | 16 |
| Furdyna A, Ciurzyński M, Roik M, Paczyńska M, Wretowski D, Jankowski K, et al. Management of high risk pulmonary embolism - a single center experience. Folia medica Cracoviensia. 2018;58(4):75-83. | 32 | Shock or hypotension on admission with systolic blood pressure below 90 mm Hg and signs of peripheral hypoperfusion, if not caused by new arrhythmia, sepsis, or hypovolemia. | Thrombolysis, percutaneous embolectomy, or anticoagulation. | Retrospective, single-arm, single-center, non-randomized study | Poland | NS | 2006-2017 | 71 ± 18 | 13 |
| George B, Parazino M, Omar HR, Davis G, Guglin M, Gurley J, et al. A retrospective comparison of survivors and non-survivors of massive pulmonary embolism receiving veno-arterial extracorporeal membrane oxygenation support. Resuscitation. 2018;122:1-5. 10.1016/j.resuscitation.2017.11.034 | 32 | Systolic blood <90 mmHg for >15 min, requiring vasopressors, and/or demonstrating evidence of organ dysfunction. | ECMO and anticoagulation, with either systemic thrombolysis, catheter directed thrombolysis, surgical thrombectomy or aspiration thrombectomy. | Retrospective, single-arm, single-center, non-randomized study | US | NS | 2012-2015 | Survived: 56 (IQR: 43-70) Died: 56 (IQR: 50-62) | 17 |
| Hartman AR, Manetta F, Lessen R, Pekmezaris R, Kozikowski A, Jahn L, et al. Acute surgical pulmonary embolectomy: a 9-year retrospective analysis. Texas Heart Institute journal. 2015;42(1):25-9. 10.14503/thij-13-3877 | 24 | Severe, globally hypokinetic RV dysfunction on TTE, and CTA–documented PE, with either a large clot burden in the main pulmonary arteries or a saddle embolism. Unstable cohort only (24/96 patients), with vasopressors for SBP <90mmHg. | Surgical pulmonary embolectomy | Retrospective, single-arm, single-center, non-randomized study | US | NS | 2003-2011 | NS | NS |
| Kjaergaard B, Kristensen JH, Sindby JE, de Neergaard S, Rasmussen BS. Extracorporeal membrane oxygenation in life-threatening massive pulmonary embolism. Perfusion. 2019;34(6):467-74. 10.1177/0267659119830014 | 38 | Cardiac arrest or unstable circulation with systolic blood pressure < 90 mmHg for at least 15 minutes. | ECMO with thrombolysis, heparin or surgical thrombectomy. | Retrospective, single-arm, single-center, non-randomized study | Denmark | 12 | 2004-2017 | 55 (19-81) | 18 |
| Kuo WT, Banerjee A, Kim PS, DeMarco FJ, Jr., Levy JR, Facchini FR, et al. Pulmonary Embolism Response to Fragmentation, Embolectomy, and Catheter Thrombolysis (PERFECT): Initial Results From a Prospective Multicenter Registry. Chest. 2015;148(3):667-73. 10.1378/chest.15-0119 | 28 | Sustained hypotension with systolic BP <90 mm Hg for at least 15 min or requiring inotropic support | Catheter-directed mechanical or pharmaco-mechanical thrombectomy and/or catheter-directed thrombolysis through low-dose hourly drug infusion with tissue plasminogen activator or urokinase. | Retrospective, single-arm, multi-center, non-randomized study | Europe and US | NS | 2011-2014 | NS | NS |
| Lehnert P, Møller CH, Mortensen J, Kjaergaard J, Olsen PS, Carlsen J. Surgical embolectomy compared to thrombolysis in acute pulmonary embolism: morbidity and mortality. European journal of cardio-thoracic surgery: official journal of the European Association for Cardio-thoracic Surgery. 2017;51(2):354-61. 10.1093/ejcts/ezw297 | 64 | Documented systolic blood pressure lower than 90mmHg or a systolic blood pressure drop of 40mmHg for at least 15 min at the time of diagnosis as defined in the ESC guidelines. | Surgical embolectomy or thrombolysis | Retrospective, single-arm, single-center, non-randomized study | Denmark | 31 (8 – 133) | 2008-2014 | NS | 26 |
| Meneveau N, Guillon B, Planquette B, Piton G, Kimmoun A, Gaide-Chevronnay L, et al. Outcomes after extracorporeal membrane oxygenation for the treatment of high-risk pulmonary embolism: a multicentre series of 52 cases. European heart journal. 2018;39(47):4196-204. 10.1093/eurheartj/ehy464 | 180 | Diagnosis of PE was in accordance with the European Society of Cardiology guidelines | Without ECMO: Fibrinolysis, embolectomy, or heparin alone. With ECMO: ECMO alone or with thrombolysis, or surgical embolectomy. | Retrospective, two-arm, multi-center, non-randomized study | France | 3 | 2014-2015 | NS | 96 |
| Minakawa M, Fukuda I, Miyata H, Motomura N, Takamoto S, Taniguchi S, et al. Outcomes of Pulmonary Embolectomy for Acute Pulmonary Embolism. Circulation journal: official journal of the Japanese Circulation Society. 2018;82(8):2184-90. 10.1253/circj.CJ-18-0371 | 63 | Patients in refectory shock (SBP <80 mmHg and/or cardiac index <1.8 L/min/m^2^) | Surgical pulmonary embolectomy. | Retrospective, single-arm, multi-center, non-randomized study | Japan | NS | 2008-2014 | NS | NS |
| Moon D, Lee SN, Yoo KD, Jo MS. Extracorporeal membrane oxygenation improved survival in patients with massive pulmonary embolism. Annals of Saudi medicine. 2018;38(3):174-80. 10.5144/0256-4947.2018.174 | 23 | Systolic blood pressure less than 90 mm Hg that did not respond to fluid supply and required a vasopressor agent. | Systemic thrombolysis (N=5), anticoagulation (N=4), anticoagulation with ECMO (N-13), or systemic thrombolysis with ECMO (N=1) | Retrospective, single-arm, single-center, non-randomized study | Korea | 3 | 2004-2010 | 65±15 (stage 1); 54±18 (stage 2) | 4 |
| Munakata R, Yamamoto T, Hosokawa Y, Tokita Y, Akutsu K, Sato N, et al. Massive pulmonary embolism requiring extracorporeal life support treated with catheter-based interventions. International heart journal. 2012;53(6):370-4. 10.1536/ihj.53.370 | 10 | Refractory shock (1/10) or cardiac arrest (9/10). | ECMO and heparin with; I.V. thrombolytics, catheter directed thrombolysis, embolus fragmentation, or thrombectomy. | Retrospective, single-arm, single-center, non-randomized study | Japan | NS | 1992-2008 | Range 23-86 | 2 |
| Neely RC, Byrne JG, Gosev I, Cohn LH, Javed Q, Rawn JD, et al. Surgical Embolectomy for Acute Massive and Submassive Pulmonary Embolism in a Series of 115 Patients. The Annals of thoracic surgery. 2015;100(4):1245-51; discussion 51-2. 10.1016/j.athoracsur.2015.03.111 | 49 | Systolic blood pressure less than 90 mm Hg for at least 15 min or requiring inotropic support. | Surgical pulmonary embolectomy. | Retrospective, single-arm, single-center, non-randomized study | US | NS | 1999-2013 | NS | NS |
| Niwa A, Nakamura M, Harada N, Musha T. Observational investigation of thrombolysis with the tissue-type plasminogen activator monteplase for acute pulmonary embolism in Japan. Circulation journal: official journal of the Japanese Circulation Society. 2012;76(10):2471-80. 10.1253/circj.cj-12-0091 | 289 | Massive group defined as unstable hemodynamics, shock in 100%, syncope in 100%. Cardiopulmonary arrest/collapse group were also included. | Thrombolysis with percutaneous interventions, surgical embolectomy, percutaneous cardiopulmonary support, anticoagulation | Prospective, single-arm, single-center, non-randomized study | Japan | NS | 2005-2008 | Median: 67 | 78 |
| Pasrija C, Shah A, George P, Kronfli A, Raithel M, Boulos F, et al. Triage and optimization: A new paradigm in the treatment of massive pulmonary embolism. The Journal of thoracic and cardiovascular surgery. 2018;156(2):672-81. 10.1016/j.jtcvs.2018.02.107 | 56 | Systolic blood pressure<90 mm Hg for at least 15 minutes or the need for inotropic support. | Surgical pulmonary embolectomy (control group; N=27).  VA-ECMO (protocol group; N=29) with surgical pulmonary embolectomy (N=14) or anticoagulation (N=15). | Retrospective, single-arm, single-center, non-randomized study | US | 17 (median) | 2010-2017 | Historical control: 60 (IQR: 45-67) Protocol: 50 (IQR: 41-59) | 30 |
| Roncon L, Zuin M, Casazza F, Becattini C, Bilato C, Zonzin P. Impact of syncope and pre-syncope on short-term mortality in patients with acute pulmonary embolism. European journal of internal medicine. 2018;54:27-33. 10.1016/j.ejim.2018.04.004 | 77 | Persistent systolic blood pressure (SBP)≤90 mmHg or a pressure drop of ≥40 mmHg for >15 min.. | Thrombolysis (N=36) (other treatments not specified). | Prospective, single-arm, single-center, non-randomized study | Italy | NS | 2006-2010 | Syncope 75±12; Presyncope80±11 | 27 |
| Secemsky E, Chang Y, Jain CC, Beckman JA, Giri J, Jaff MR, et al. Contemporary Management and Outcomes of Patients with Massive and Submassive Pulmonary Embolism. The American journal of medicine. 2018;131(12):1506-14.e0. 10.1016/j.amjmed.2018.07.035 | 46 | Sustained (>15minutes) hypotension (systolic blood pressure <90mmHg) or any period of pulselessness. | Systemic anticoagulation with IVC filter, catheter-directed thrombolysis, ECMO, or surgical embolectomy. | Prospective, single-arm, single-center, non-randomized study | US | 12 | NS | 63±14 | 20 |
| Senturk A, Ucar EY, Berk S, Ozlu T, Altinsoy B, Dabak G, et al. Should Low-Molecular-Weight Heparin be Preferred Over Unfractionated Heparin After Thrombolysis for Severity Pulmonary Embolism? Clinical and applied thrombosis/hemostasis: official journal of the International Academy of Clinical and Applied Thrombosis/Hemostasis. 2016;22(4):395-9. 10.1177/1076029614564863 | 186 | References ESC guidelines for defining the high-risk subset and specifies “shock or hypotension.”. | Thrombolytic therapy followed by low-molecular-weight heparin or unfractionated heparin. | Prospective, two-arm, multi-center, non-randomized study | Turkey | 1 | 2013 | NS | NS |
| Sharifi M, Berger J, Beeston P, Bay C, Vajo Z, Javadpoor S. Pulseless electrical activity in pulmonary embolism treated with thrombolysis (from the "PEAPETT" study). The American journal of emergency medicine. 2016;34(10):1963-7. 10.1016/j.ajem.2016.06.094 | 23 | Cardiac arrest, pulseless electrical activity. | Tissue plasminogen activator followed by heparin. | Retrospective, single-arm, single-center, non-randomized study | US | 22±3 | 2013-2015 | 72±5 | 9 |
| Shiomi D, Kiyama H, Shimizu M, Yamada M, Shimada N, Takahashi A, et al. Surgical embolectomy for high-risk acute pulmonary embolism is standard therapy. Interactive cardiovascular and thoracic surgery. 2017;25(2):297-301. 10.1093/icvts/ivx091 | 31 | Hemodynamically unstable; massive clots in bilateral pulmonary arteries or unilateral pulmonary artery occlusion with a floating clot in the main pulmonary artery or right atrium and right ventricular dilatation in transthoracic echocardiography. | Surgical pulmonary embolectomy. All patients received anticoagulation (unfractionated heparin) post-operatively. | Retrospective, two-arm, single-center, non-randomized study | Japan | 48±36 | 2004-2014 | 58±12 (Range: 26-78) | 11 |
| Ucar EY, Araz O, Akgun M, Meral M, Kalkan F, Saglam L, et al. Low-molecular-weight heparin use with thrombolysis: is it effective and safe? Ten years' clinical experience. Respiration; international review of thoracic diseases. 2013;86(4):318-23. 10.1159/000346203 | 107 | Sustained hypotension (systolic arterial pressure <90 mm Hg or a drop in systolic arterial pressure of at least 40 mm Hg for at least 15 min) and cardiogenic shock | Subcutaneous low molecular weight heparin with thrombolytic therapy. | Retrospective, two-arm, single-center, non-randomized study | Turkey | NS | 2000-2010 | 63±15 | 45 |
| Wang L, Xu Y, Zhang W, Lu W, Chen M, Luo J. Early interventional therapy for acute massive pulmonary embolism guided by minimally invasive hemodynamic monitoring. International journal of clinical and experimental medicine. 2015;8(8):14011-7. | 40 | Systolic blood pressure < 90 mmHg for at least 15 minutes or requiring inotropic support, not due to a cause other than PE.) | Systemic thrombolysis followed by monitoring with (a) the invasive hemodynamic Vigileo/FloTrac System (N=20; experimental group) or (b) echocardiography (N=20; control group). | Prospective, two-arm, single-center, randomized study | China | NS | 2010-2014 | Experimental group: 62±6 Control group: 61±6 | 21 |
| BP, Blood Pressure; CTA, Computed Tomographic Angiography; ECMO, Extracorporeal Membrane Oxygenation; ESC, European Society of Cardiology; IQR, Interquartile Range; I.V., Intravenous; IVC, Inferior Vena Cava; MPE, Massive Pulmonary Embolism; NS, Not Specified; PE, Pulmonary Embolism; RV, Right Ventricle; SBP, Systolic Blood Pressure; TTE, Transthoracic Echocardiography; VA-ECMO, Veno-Arterial ECMO  *Follow-up was reported in months as either mean ± standard deviation, median (range), or maximum (X), unless otherwise stated.  †Age was reported in years as mean ± standard deviation, median (range) unless otherwise stated. | | | | | | | | | |

| Supplemental Table 2. Mortality Outcomes for the Studies- ON-LINE ONLY | | | |
| --- | --- | --- | --- |
| First Author | In-Hospital Mortality | 30 Day ACM | PE Related Mortality |
| Avgerinos et al. 2018 | 15/90, 16.6% | NS | NS |
| Barrett et al. 2010 | SE: 6/9, 66.6% | NS | SE: 4/9, 44.4% |
|  | TL: 6/10, 60.0% |  | TL: 5/10, 50.0% |
|  | AC: 5/14, 35.7% |  | AC: 5/14, 35.7% |
| Bottega et al. 2020 | NS | NS | NS |
| Carvalho et al. 2010 | 7/16, 43.8% | 7/16, 43.8% | NS |
| Cho et al. 2016 | NS | TL: 7/19, 36.8% | NS |
|  |  | SE: 4/26, 15.4% |  |
| Corsi et al. 2017 | NS | NS | NS |
| de Winter et al. 2019 | NS | 16/33, 48.5% | 1/33, 3% |
| Furdyna et al. 2018 | NS | NS | 16/32, 50% |
| George et al 2018 | 15/32, 46.9% | NS | NS |
| Hartman et al. 2015 | NS | 3/24, 12.5% | NS |
| Kjaergaard et al. 2019 | 10/38, % | 13/38, % | NS |
| Kuo et al. 2015 | 4/28, 14.3% | NS | NS |
| Lehnert et al. 2017 | NS | 15/64, 23.4% | NS |
| Meneveau et al. 2018 | NS | 87/180, 48.3% | NS |
| Minakawa et al. 2018 | 23/63, 36.5%* | NS | NS |
| Moon et al. 2018 | Without ECMO:7/9,77.8% | Without ECMO:7/9,77.8% | NS |
|  | ECMO: NS | ECMO: 8/14, 57.1% |  |
| Munakata et al. 2012 | 3/10, 30.0%† | 3/10, 30% | NS |
| Neely et al. 2015 | 5/49, 10.2%* | NS | NS |
| Niwa et al. 2012 | NS | NS | NS |
| Pasrija et al. 2018 | Control group: 5/27, 18.5% | NS | NS |
|  | Protocol group: 1/29, 3.4% | NS | NS |
| Roncon et al. 2018 | NS | Syncope: 12/47, 25.5% | NS |
|  | NS | Presyncope: 13/30, 43.3% | NS |
| Secemsky et al. 2019 | 15/46, 32.6% | 16/46, 34.8% | 9/20, 45.0% |
| Senturk et al. 2016 | NS | 30/186, 16.1% | NS |
| Sharifi et al. 2016 | 2/23, 8.7% | NS | NS |
| Shiomi et al. 2017 | 4/31, 12.9% | NS | NS |
| Ucar et al. 2013 | 18/107, 16.8% | NS | NS |
| Wang et al. 2015 | NS | Experimental group: 0/20, 0% | NS |
|  |  | Control group: 1/20, 5.0% |  |
| AC, Anticoagulation; ACM, All-Cause Mortality; FU, Follow-Up; NA, Not Applicable; NS, Not Specified; PE, Pulmonary Embolism; SE, Surgical Embolectomy; TL, Thrombolytic Therapy  *Operative mortality was reported. Only patients in refractory shock were included in the analysis.  †All patients died within 15 hours of the procedure. | | | |

| Supplemental Table 3. Secondary Outcome Measures by Publication | | | | | | | | | |
| --- | --- | --- | --- | --- | --- | --- | --- | --- | --- |
| First Author | Group | Treatment Crossover | Clinical Deterioration | BARC 3b Bleeding | Intracranial Hemorrhage | Major Bleeding | Stroke | Renal Dysfunction | Sepsis |
| Avgerinos et al. 2018 |  | - | - | 24/90, 26.6% | 5/90, 5.5% | 24/90, 26.6% | 5/90, 5.5% | - | - |
| Barrett et al. 2010 |  | - | - | - | - | - | - | - | - |
| Bottega et al. 2020 |  | - | - | - | - | 3/13, 23.1% | - | - | - |
| Carvalho et al. 2010 |  | - | - | - | - | 2/16, 12.5% | - | 2/16, 12.5% |  |
| Cho et al. 2016 |  | 4/19, 21.1% | - | - | - | - | - | - | - |
| Corsi et al. 2017 |  |  | - | - | - | - | 4/17, 23.5% | - | - |
| de Winter et al. 2019 |  | 8/33, 24.2% | - | - | - | 12/33, 36.4% | - | - | - |
| Furdyna et al. 2018 |  | - | - | 5/32, 15.6% | - | 9/32, 28.1% | - | - | - |
| George et al 2018 |  | - | 5/32, 15.6% | - | - | - | - | - | - |
| Hartman et al. 2015 |  | - | - | - | - | - | - | - | - |
| Kjaergaard et al. 2019 |  | - | - | - | - | - | - | - | - |
| Kuo et al. 2015 |  | - | - | 0/28, 0.0% | 0/28, 0.0% | 0/28, 0.0% | 0/28, 0.0% | - | - |
| Lehnert et al. 2017 |  | - | - | - | - | 1/64, 1.6% | - | - | - |
| Meneveau et al. 2018 |  | - | - | - | - | 28/180, 1.5% | - | - | - |
| Minakawa et al. 2018 |  | - | - | - | - | - | - | - | - |
| Moon et al. 2018 |  | - | - | 7/14, 50.0% | - | 7/14, 50.0% | - | - | - |
| Munakata et al. 2012 |  | - | - | 2/10, 20.0% | - | 2/10, 20.0% | - | - | - |
| Neely et al. 2015 |  | - | - | 1/49, 2.0% | - | 1/49, 2.0% | 2/49, 4.1% | 3/49, 6.1% | 3/49, 6.1% |
| Niwa et al. 2012 |  | - | - | - | 8/289, 2.8% | 29/289, 10.0% | - | - | - |
| Pasrija et al. 2018 | Control group | - | - | 3/27, 11.1% | - | 3/27, 11.1%  4/29, 13.8% | 0/27, 0.0% | 4/27, 14.8% | 1/27, 3.7% |
|  | Protocol Group |  |  | 4/29, 13.8% |  |  | 0/29, 0.0% | 4/29, 13.8% | 1/29, 3.4% |
| Roncon et al. 2018 |  | - | - | - | - | - | - | - | - |
| Secemsky et al. 2019 |  | - | - | 11/46, 23.9% | 2/46, 4.3% | 11/46, 23.9% | - | - | - |
| Senturk et al. 2016 |  | - | - | 10/186, 5.4% | - | 10/186, 5.4% | - | - | - |
| Sharifi et al. 2016 |  | - | - | 0/23, 0.0% | - | 0/23, 0.0% | - | - | - |
| Shiomi et al. 2017 |  | - | - | - | - | - | - | - | - |
| Ucar et al. 201 |  | - | - | 4/107, 3.8% | - | 4/107, 3.8% | - | - | - |
| Wang et al. 2011 | Experimental Group | 12/20, 60.0% | - | - | - | - | - | - | - |
|  | Control Group | 4/20, 20.0% |  |  |  |  |  |  |  |
